# Rational stabilization of complex proteins: a divide and combine approach

**DOI:** 10.1038/srep09129

**Published:** 2015-03-16

**Authors:** Emilio Lamazares, Isabel Clemente, Marta Bueno, Adrián Velázquez-Campoy, Javier Sancho

**Affiliations:** 1 Biocomputation and Complex Systems Physics Institute (BIFI)-Joint Unit BIFI-IQFR (CSIC), Universidad de Zaragoza, Zaragoza, Spain; 2 Departamento de Bioquímica y Biología Molecular y Celular, Facultad de Ciencias, Universidad de Zaragoza, Zaragoza, Spain; 3 Fundación ARAID, Gobierno de Aragón, Spain

## Abstract

Increasing the thermostability of proteins is often crucial for their successful use as analytic, synthetic or therapeutic tools. Most rational thermostabilization strategies were developed on small two-state proteins and, unsurprisingly, they tend to fail when applied to the much more abundant, larger, non-fully cooperative proteins. We show that the key to stabilize the latter is to know the regions of lower stability. To prove it, we have engineered apoflavodoxin, a non-fully cooperative protein on which previous thermostabilizing attempts had failed. We use a step-wise combination of structure-based, rationally-designed, stabilizing mutations confined to the less stable structural region, and obtain variants that, according to their van't Hoff to calorimetric enthalpy ratios, exhibit fully-cooperative thermal unfolding with a melting temperature of 75°C, 32 degrees above the lower melting temperature of the non-cooperative wild type protein. The ideas introduced here may also be useful for the thermostabilization of complex proteins through formulation or using specific stabilizing ligands (e.g. pharmacological chaperones).

Proteins are important analytic, synthetic and therapeutic tools^1^,^2^,^3^. Controlling the stability of proteins is crucial for their successful manufacture, storage, combination, administration and, in general, for their use *ex vivo* or *in vivo* (www.nist.gov/mml/bmd/biomanufacturing.cfm). The concept of protein stability may refer to different things, such as stability towards chemical alteration^4^, kinetic stability towards transformation into non-native conformations^5^,^6^, or the thermodynamic stability of the native form, which is governed by the equilibrium constant with the ensemble of denatured conformations^7^,^8^. In many cases, increasing the conformational stability of a protein is beneficial as it brings concomitant increases in kinetic stability or chemical stability. A primary synthetic tool for protein stabilization is mutagenesis, which can be implemented in directed evolution methods based in generating diversity and selecting the more stable variants^9^,^10^, or through rational design of specific mutations, often based in analysis of the protein three-dimensional structure and in understanding the interactions governing protein folding^11^,^12^. Many rational strategies have been developed to stabilize proteins, including α-helix stabilization^13^, charge optimization^14^,^15^, cavity filling^16^,^17^, disulfide bond engineering^18^,^19^, replacement of hydrophobic external residues^20^,^21^ or of polar buried residues^22^, neutralization of hydrogen bonds^23^, denatured state entropy reduction^24^, or structure-guided consensus^25^,^26^. Although, admittedly, any specific mutation based on any of those strategies may fail, implementation of several such mutations in a given protein is expected to yield in most cases a more stable variant.

For convenience, the development and testing of rational protein stabilization strategies has heavily relied in the engineering of small model proteins of less than 200 residues and typically displaying simple two-state unfolding equilibria towards denaturant-induced unfolding or towards thermal denaturation^27^,^28^. In this endeavor, the chief design principle has consisted in introducing amino acid replacements that either lower the enthalpy of the native state relative to the denatured one, or lower the entropy of the denatured state relative to the native one, on the assumption that the denatured state is essentially unstructured and devoid of the interactions observed in the native structure. However, small proteins constitute a small fraction of proteomes. According to the 01-Oct-14 release of UniProt/SwissProt (http://web.expasy.org/docs/relnotes/relstat.html) the average protein length is 355 residues. Proteins of that size are expected to deviate from the two-state behavior and to populate partly unfolded conformations in addition to the native and fully unfolded ones. Examples of three-state unfolding equilibria abound even among small, one-domain proteins (e.g. in the thermal unfolding of 149-residue staphylococcal nuclease mutants^29^, in 104-residue onconase^30^ or in 169-residue apoflavodoxin^31^). In general, non-fully cooperative proteins will contain at least two structural regions of different stability that will often unfold sequentially (Figure 1). In many cases, the unfolding of the less stable region will signal the loss of the biological activity, and the population of a partly unfolded intermediate that may be prone to aggregation. For that majority of large proteins that are unlikely to display full unfolding cooperativity, using the common strategies of rational design without previous identification of the less stable region may be very disappointing^15^. The reason is that, unlike for denatured states, the structure of equilibrium intermediates cannot be easily anticipated, and assuming homogeneously unstructured intermediates is not realistic.

The apoflavodoxin from *Anabaena* PCC 7119 is a small 169-residue, monodomain protein displaying two-state urea denaturation and a three-state thermal unfolding^31^,^32^. Its thermal intermediate is characterized by a severe loss of native structure at the FMN cofactor binding site^33^,^34^,^35^ and it is considered, for this reason, to be non-functional. At 25.0°C, in native conditions, the thermal intermediate represents around 7% of the protein population^35^. Therefore, apoflavodoxin can be considered a three-state protein. Previous attempts to rationally increase its thermostability by introducing charge reversal mutations that had greatly increased the global conformational stability at 25°C unexpectedly failed because the design had ignored the intrinsic complexity of non-two state proteins^15^. After having delineated through experimental^33^,^34^,^35^ and computational work^36^ the two stability regions of this protein, we show here how a complex protein can be thermostabilized by specifically administering rational strategies to its less stable region. By a step-wise combination of rationally designed individual stabilizing mutations we have increased the lower melting temperature of the protein by 32 degrees and converted it from three-state to a fully cooperative two-state protein, according to its van't Hoff to calorimetric enthalpy ratio.

## Experimental section

### Design of mutants

All mutants have been designed from inspection and analysis of the x-ray structure of wild type (WT) apoflavodoxin^37^ from *Anabaena* PCC 7119 (pbd id 1ftg) and the solution structure^34^ of the F98N apoflavodoxin mutant (pbd id 2kqu), which closely corresponds to the structure of the partly unfolded apoflavodoxin thermal intermediate^33^,^34^,^35^,^36^ (Figure 2).

Apoflavodoxin contains two structural regions of different stability^33^. Eleven single stabilizing mutations (Table 1) have been designed in the less stable region or at the interface of the two regions with the aim of specifically increasing the lower melting temperature: *T*_1_. The rationale behind the mutations is the following:

- I59A and I92A: replacement of highly exposed hydrophobic residues by shorter ones. The ProtSA server^38^ was used to identify those residues as highly exposed in the native structure. Ile59 is more exposed in the native state than in the unfolded ensemble, while Ile92 is similarly exposed in either state.
- Q99A and R155L: replacement of poorly exposed, polar neutral or charged residues by non polar ones. The ProtSA server^38^ was used to identify those residues by the low solvent exposure of their polar or charged side chain atoms in the folded state (0 and 8 Å^2^, respectively).
- E61K and D126K: replacement of negatively charged residues in proximity to other negative charges by positively charged residues so that electrostatic repulsion in the native state is decreased. The electrostatic unfavorable interactions of Glu61 and Asp126 were calculated using electrostatic simulations^14^,^15^.
- D100N: replacement of a hydrogen bonded aspartate residue by a neutral, isosteric asparagine one. The stabilizing effect of converting charged hydrogen bonds into neutral ones was previously reported^23^.
- G87V and A142V: replacement of small residues located near a small protein cavity by bulkier ones so that the cavity is filled, with gain in van der Waals interactions and hydrophobic effect. This strategy was successfully tested previously in other cavities of the same protein^17^.
- S121P and D129A: replacement of two solvent exposed residues by residues more frequent in structurally equivalent positions of homologous flavodoxins. This consensus strategy has been reported to be effective in other proteins^25^,^26^.

On the other hand, two charge reversal mutations located in the stable flavodoxin region (E20K, E72K), and previously reported to specifically increase the higher melting temperature of the protein: *T_2_*^15^, have now been combined with mutation D126K (located at the less stable region, see above) to construct a triple mutant, termed 3M, which is significantly more stable than WT apoflavodoxin. Both WT and 3M apoflavodoxins have been used as starting points to test the feasibility of introducing and combining stabilizing mutations at the two stability regions in order to rationally push the lower melting temperature of this three-state protein towards higher values. The specific stabilizing mutations and combinations introduced and tested are described in Tables 1 and 2.

### Mutagenesis, protein expression, purification, and spectroscopic characterization by circular dichroism

All mutated flavodoxin genes were synthesized by site-direct mutagenesis using the Mutagenex Inc company service (www.mutagenex.com). The sequence of some of the mutated genes, randomly selected, was verified by additional sequencing using different primers, and always coincided with the reported one (not shown). Mutated genes were introduced in the pTrc99A plasmid^39^ and expressed in BL21 *E. coli* cells as described^40^. The flavodoxin variants were purified by combining ammonium sulfate precipitation with ionic exchange in DEAE sepharose, and the corresponding apoflavodoxins were obtained by removal of the FMN cofactor by TCA precipitation of the protein moiety, as described^32^.

Circular dichroism (CD) spectra were recorded at 25.0 ± 0.1°C in a thermostated Chirascan apparatus from Applied Photophysics. Near-UV CD spectra were recorded from 260 to 310 nm using 20 μM protein solutions dissolved in 50 mM MOPS buffer, pH 7.0 with a 1-cm path length cuvette. Far-UV CD spectra were recorded from 200 to 250 nm with protein solutions of the same concentration dissolved in 5 mM MOPS buffer, pH 7.0 with 15 mM NaCl and a 1-mm cuvette.

### Thermal denaturation by absorbance, fluorescence and far-UV and near-UV CD, and their global analysis

Apoflavodoxin displays a three-state thermal denaturation mechanism according to both spectroscopic and calorimetric analysis^15^,^31^,^33^. The three-state model used and the assumptions made to analyze the apoflavodoxin thermal unfolding data has been recently reviewed in detail^8^. Briefly, the spectroscopy signal of a family of thermal unfolding curves corresponding to the same protein variant and recorded using different spectroscopic techniques is globally fitted to equation (1)
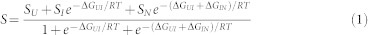
where *S* is the protein signal at a given temperature (T), *S_U_*, *S_I_* and *S_N_* are the signals of protein solutions of equal concentration of either unfolded, intermediate or native protein respectively, and Δ*G_UI_* and Δ*G_IN_* are the free energy differences of the equilibria between the unfolded and intermediate conformations (residual stability) or between the intermediate and the native conformation (relevant stability). Those free energies are approximated by the integrated Gibbs-Helmholtz equation:
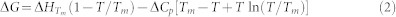
where *T_m_*, Δ*H* and Δ*C_p_* are the melting temperature, enthalpy change and heat capacity change of the specific equilibrium.

The thermostability of forty apoflavodoxin variants has been determined in 50 mM MOPS, pH 7.0 as follows. For each variant, four unfolding curves in 50 mM MOPS, pH 7.0, from approximately 8 to 94°C, were monitored by far-UV CD (222 nm; 20 μM protein, 1-mm path length), near-UV CD and near-UV absorbance (290 nm; 20 μM protein, 4-mm path length) and fluorescence emission (excitation at 280 nm; ratio of 320/360 nm emission; 2 μM protein). Although, using fluorescence emission ratios to minimize the effect of temperature in curve baselines is not generally advised^8^, the inaccuracy introduced in individual *T*_m_s is minimized when differences in *T_m_* values (Δ*T_m_*) are calculated^41^. The four unfolding curves obtained for each variant were roughly normalized between 0 and 1 and then globally fitted to a three-state equation using Origin 8.0 (OriginLab Corporation) and MLAB software (Civilized software). The global fitting of the four curves provides, for each mutant protein, a single set of Δ*H_1_*, *T_1_*, Δ*C_p1_*, Δ*H_2_*, *T_2_*, and Δ*C_p2_* values plus specific sets of spectroscopic parameters for each curve^8^,^33^. The melting temperatures (*T_1_* and *T_2_*) and enthalpy changes (Δ*H_1_* and Δ*H_2_*) corresponding to the two unfolding transitions of each mutant are reported in Table 1 (WT series) and Table 2 (3M series). The values obtained in global spectroscopic fits for the changes in heat capacity of the transitions are not accurate^42^ and will not be reported. Discrimination between two- and three-state models was performed based on the: 1) visual inspection of fitting analysis (fits and residuals); and 2) comparison of the chi-square values. Only variants whose set of unfolding curves were not superimposable (i.e. did not provide essentially the same *T*_m_ and Δ*H* values when individually analyzed) were subjected to three-state analysis.

### Thermal denaturation by differential scanning calorimetry (DSC)

The Δ*H_1_*, *T_1_*, Δ*C_p1_*, Δ*H_2_*, *T_2_*, and Δ*C_p2_* values of wild-type and apoflavodoxin mutants were also measured with a high-precision differential scanning VP-DSC microcalorimeter (MicroCal LLC, Northampton, MA). All samples were degassed and loaded into the cells, avoiding bubble formation. The heat capacity of apoflavodoxin was measured as a function of temperature. The baseline of the instrument was recorded before the experiments with both cells filled with buffer. Thermal denaturation scans, from 10 to 110°C, were performed using 40 μM protein solutions in 50 mM MOPS, pH 7.0 with a scanning rate of 1 °C/min. DSC data analysis was performed by using a two-state or a three-state model^43^,^44^ implemented in the Origin 7.0 software package. Discrimination between two- and three-state models was performed based on the: 1) visual inspection of fitting analysis (fits and residuals); 2) comparison of the chi-square values; and 3) comparison of the van't Hoff and calorimetric enthalpies.

### Chemical denaturation and data analysis

Protein solutions were prepared mixing 900-μL urea solutions (from 0 M to 7.2 M) with 100 μL of 20 mM apoprotein solutions in 500 mM MOPS, pH 7.0^32^. All samples were equilibrated at 25°C for 30 min. Protein unfolding was followed by fluorescence emission at 320 nm with excitation at 280 nm (25.0 ± 0.1°C) using a thermostated Cary Eclipse Fluorescence Spectrophotometer. Apoflavodoxin urea unfolding follows a simple two-state mechanism^32^. Thus, the curves were analyzed by the linear extrapolation method^45^ using a two-state equation implemented in Origin 8.0 (Origin Lab Corporation), essentially as described^17^.

### Calculation of stability differences: relevant and residual stabilization

The difference in stability between a wild type protein and a mutant thereof (Δ*G^wt^-* Δ*G^mut^)* is best calculated using the following simplified equation^8^,^46^ that holds in the proximity of the transition temperature of WT:
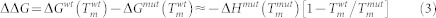


For a three-state thermal unfolding equilibrium (N↔I↔U) such as that of apoflavodoxin^31^, the global stability of the protein (Δ*G_NU_*) is divided in two terms:
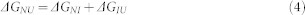
where Δ*G_NI_* represents the “relevant” stability of the native state relative to the intermediate^54^ while Δ*G_IU_* is the “residual stability” of the intermediate relative to the fully unfolded state. The term “relevant stability” has been chosen to indicate that the intermediate in non-functional, as suggested by the disruption of the cofactor binding site^33^,^34^,^35^. Comparison of wild type and mutant three-state proteins allows to calculate the changes brought about by a given mutation in both the relevant and the residual stabilities (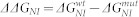; 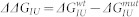. In the proximity of 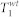, the temperature characterizing the N-to-I transition, the change in relevant stability can be approximated as:
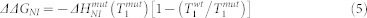
A negative value of the free energy variations defined in equation 5 indicates that the mutant is more thermostable than the wild type protein (or the appropriate reference protein) for the indicated transition.

## Results and Discussion

### Why complex proteins are more difficult to stabilize rationally than two-state proteins

Early protein stability studies showed^47^ that the native three-dimensional structure of many proteins coexists, in thermodynamic equilibrium, with a vast number of unstructured, fluctuating conformations known as the unfolded state or, more modernly, unfolded ensemble. This observation inspired the two-state model (N↔U) so widely used to quantify the stability of proteins^42^.

A two-state unfolding equilibrium indicates that the native structure is stabilized in a highly cooperative manner, the cooperativity arising from mutual strengthening of pairwise interactions due to entropic effects^48^. Although the unfolding equilibrium of many small proteins can indeed be described by the two-state model^49^, examples abound of more complex proteins that, at moderate concentrations of denaturant or solution temperatures, populate intermediate conformations at equilibrium with the native and unfolded states^30^,^50^,^51^,^52^,^53^. Given the average size of proteins, non-two state unfolding might be in fact much more common than two-state. The simplest case of a non-two-state equilibrium is that of monomeric, three-state proteins with two transitions (N↔I↔U), each characterized by a free energy difference and a specific melting temperature. For those proteins, the global conformational stability (Δ*G_NU_*) is made of two terms (Equation 4): Δ*G_NI_*, which has been termed the relevant stability of the protein to indicate that the thermal intermediate is not expected to remain functional, and Δ*G_IU_*, the residual stability of the partly unfolded intermediate^54^. In two-state proteins, global and relevant stability mean the same because there is no residual stability. Determining the stability of three-state proteins with approaches adapted from those applied to two-state ones is a fairly easy task, although it may involve acquiring more experimental data. However, stabilizing three-state proteins using the rational synthetic strategies developed over the years for thermostabilization of two-state proteins is not so easy. In fact, it poses a specific difficulty that needs to be understood in order to minimize failures.

**Figure 1:**
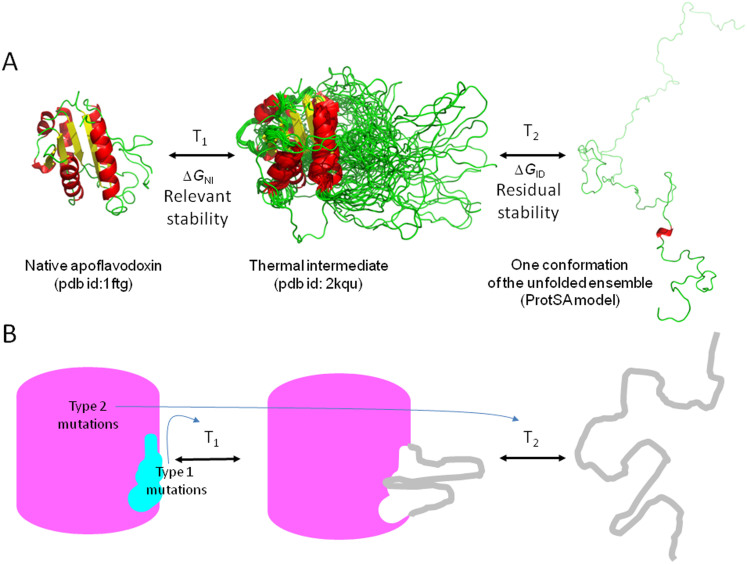
Unfolding equilibria of a three-state protein. (A) Ribbon cartoons representing the conformation of apoflavodoxin in the three states populated in its thermal unfolding equilibrium. The native state in represented by the crystal structure of the WT protein (pdb id: 1ftg); the intermediate state by the solution structure of the F98N variant (pbd id: 2kqu), and the unfolded state by one of the 2000 conformations calculated for the unfolded ensemble using the ProtSA server. The low temperature transition (*T_1_*) signals the unfolding of the less stable region leading to an equilibrium intermediate. The higher temperature transition (*T_2_*) represents the unfolding of the intermediate, leading to the unfolded state. The free energy difference between the native and the intermediate conformation (Δ*G_NI_*) is termed relevant stability of the protein while that between the intermediate and the fully unfolded conformation (Δ*G_IU_*) is termed residual stability of the protein. (B) Simplified scheme depicting a protein with two structural regions of different stability (less stable region in cyan, and more stable one in pink) and the likely effects of mutations on *T_1_* and *T_2_*. Type 1 mutations, those introduced in the unstable region or at its interface with the more stable one, will mainly modify the relevant stability of the protein. Type 2 mutations, those introduced in the more stable region, will only modify the residual stability of the protein.

Clearly, if the goal is to increase the thermal stability of a three-state protein, one should concentrate in increasing *T_1_*, the temperature at which half of the molecules remain folded and half adopt the conformation of the intermediate, rather than in increasing *T_2_*, the melting temperature of the already partly unfolded intermediate (Figure 1). Our early work in stabilization of the model protein apoflavodoxin (a single domain protein exhibiting two-state urea denaturation and three-state thermal unfolding)^31^,^32^ showed that many rationally designed mutations that successfully increased the overall stability of the protein (Δ*G_NU_*) failed to increase *T_1_*. Instead, most of the stabilization emerged as higher values of *T_2_*^15^. In practice, those apoflavodoxin variants were more stable than wild type at 25°C but not more thermostable because their *T_1_* values were the same. This paradox can be explained by the lower cooperativity exhibited by three-state proteins compared to two-state ones. Structural analysis of the apoflavodoxin equilibrium intermediate revealed that it retains the native conformation in approximately two thirds of the structure, while the other third is unfolded^33^. Thus, the single folding domain of the protein is divided into two stability structural regions: a large one with a greater thermostability governed by *T_2_*, and a smaller one that becomes unfolded at the lower temperature *T_1_*. All stabilizing mutations located in the larger, more stable region failed to increase *T_1_* and only increased the thermostability of the intermediate towards full unfolding. Conversely, all the stabilizing mutations located in the less stable region did increase *T_1_*, as initially intended^15^. The reason why complex proteins are more difficult to stabilize rationally than two-state ones is thus quite simple: if the less stable structural region is not known in advance, the probability that a designed mutation locates by chance at the more stable, larger structural region is high. Based in these observations we hypothesize that the specific increasing of *T_1_* in order to raise the relevant stability of a three-state protein requires specific intervention at the less stable structural region.

### Rational, specific stabilization of each of the two apoflavodoxin stability structural regions

**Figure 2:**
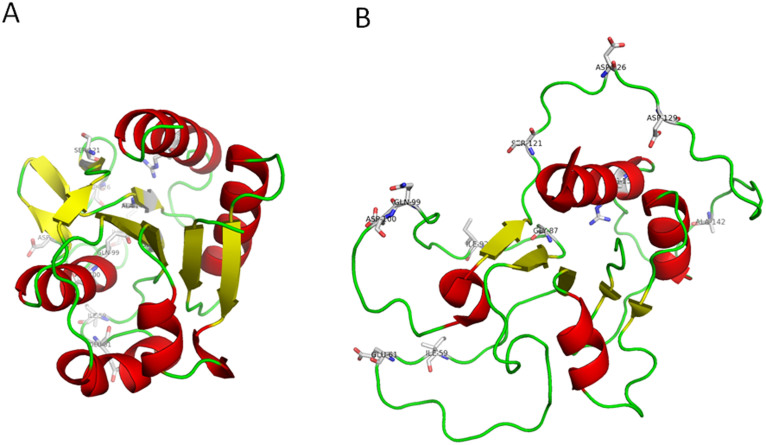
Ribbon diagrams showing the structure of native apoflavodoxin (pdb id: 1ftg) (A) and that of the apoflavodoxin thermal unfolding intermediate (B), represented by the solution structure of the F98N apoflavodoxin mutant (pbd id: 2kqu; model 1). The residues of the less stable structural region or at the interface between the two regions that have been mutated in order to increase the relevant stability of the protein are shown in sticks representation. As can be seen (panel B), those residues appear in disordered, exposed to solvent regions of the thermal unfolding intermediate.

**Figure 3:**
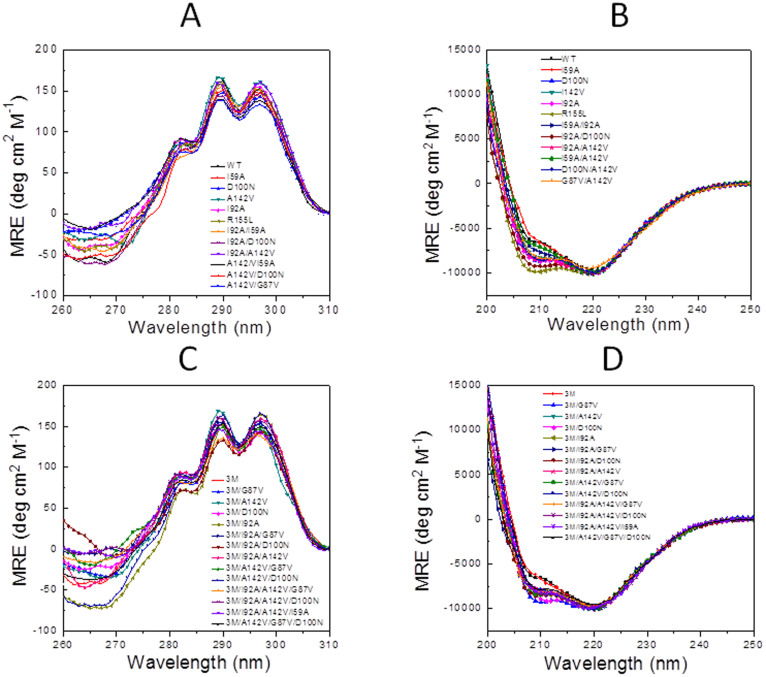
Near-UV Circular Dichroism (A, C) and far-UV Circular Dichroism (B, D) spectra of wild-type and mutants thereof (A, B) and of the 3M variant and mutants thereof (C, D) recorded at 25.0 ± 0.1°C in 50 mM MOPS buffer, pH 7.0 (near-UV spectra) or 5 mM MOPS buffer, pH 7.0 with 15 mM NaCl (far-UV spectra).

Previous experimental work based on equilibrium phi-analysis and NMR^33^,^34^ established that the less stable region of apoflavodoxin comprises segments 87-108 and 118-172, which are in contact in the three-dimensional structure. More recently, fast computational calculation of the apoflavodoxin regions exhibiting poor packing densities and forming highly polar interfaces (LIP regions^55^) identified essentially the same segments as those constituting the less stable regions of the protein. Thus, to prove our hypothesis, we have designed 11 potentially stabilizing mutations (see Experimental Section) involving residues at this less stable region of apoflavodoxin or at the interface between the two regions (Figure 2). The mutations selected are inspired by different rational strategies developed for two-state proteins over the years by our group and many others. They include shortening of highly exposed hydrophobic residues, replacement of poorly exposed polar or charged residues by neutral ones, improvement of electrostatic interactions, isosteric neutralization of hydrogen bonds, cavity filling, and replacement by more frequent residues in structurally equivalent positions. These mutations, individually or in different combinations, have been implemented in the wild type gene to produce forty apoflavodoxin variants (Tables 1 and 2) obtained with yields (4–8 mg/L) similar to those of the wild type protein (6 mg/L). To check whether the mutations alter the secondary or tertiary structure of the proteins, far-UV and near-UV CD spectra have been recorded (Figure 3). The far UV-CD spectra of the wild type series and those of mutants based on 3M apoflavodoxin (see below) are similar to those of the corresponding reference protein, although some mutants display differences at the 208 nm minima. On the other hand, the near-UV CD spectrum of apoflavodoxin is characterized by three peaks arising from tryptophan residues in the native environment that disappear when the protein is unfolded by denaturants or heat^32^. All the variants display the near-UV CD spectrum of natively folded apoflavodoxin. The CD data thus indicate that no large structural rearrangements have occurred in the 40 variants. Besides, the mutants exhibit enthalpy changes for the unfolding equilibria that are similar to those of the WT protein or of the 3M variant, (Tables 1 and 2), which also suggests the mutants retain the original tridimensional structure of apoflavodoxin.

**Figure 4:**
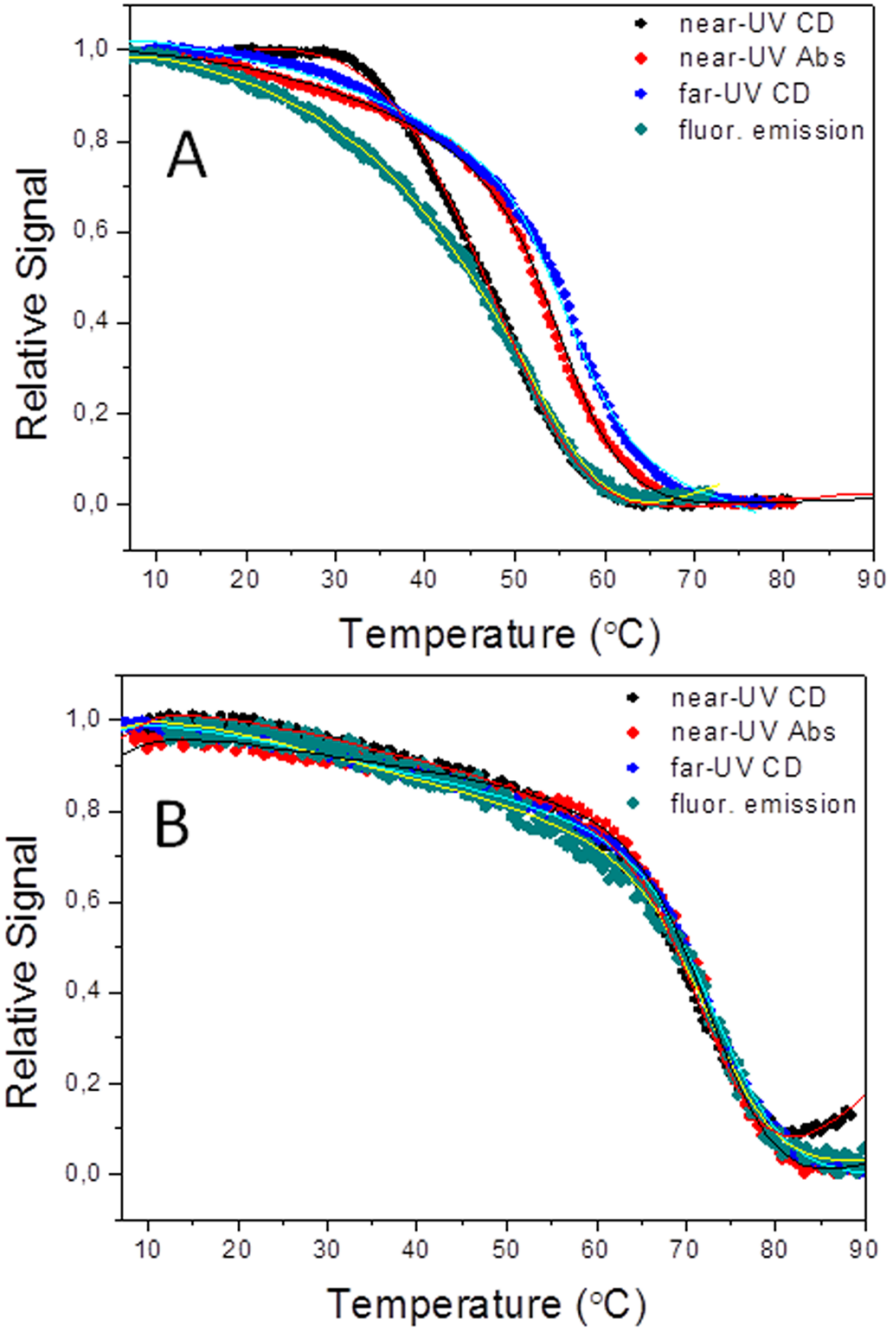
Thermal unfolding of three-state wild type apoflavodoxin (A) and of one thermostabilized two-state apoflavodoxin variant 3M/A142V/G87V/D100N (B) followed by near-UV CD (black), near-UV absorbance (red), far-UV CD (blue) and emission fluorescence (cyan). The curves are shown roughly normalized to signal spans from 0 to 1 and do not represent fractions of folded protein. For each protein, the global fit of the four curves to a three-state (WT) or a two-state model is represented by the continuous lines.

**Figure 5:**
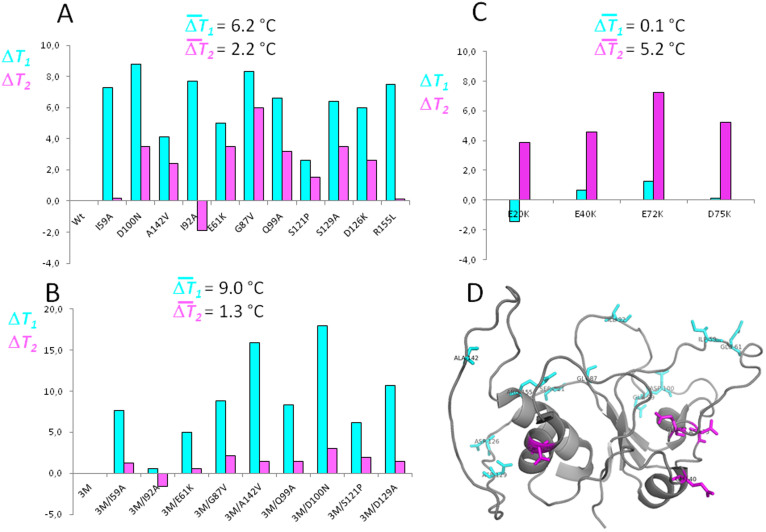
Increases in melting temperatures (Δ*T_1_* in cyan and Δ*T_2_* in magenta) observed in apoflavodoxin variants bearing mutations at either the less or the more stable structural region. Individual and average Δ*T_1_* and Δ*T_2_* relative to WT (A) or to the 3M variant (B) for mutations located in the less stable region or at the regions interface. Individual and average Δ*T_1_* and Δ*T_2_* relative to WT for mutants located at the more stable region, from Campos et al, 2004^15^ (C). Ribbon diagram of the intermediate solution structure (pbd id: 2kqu; model 1) depicting in cyan residues mutated at the less stable region of native apoflavodoxin or, in magenta, at the more stable region, which remains folded in the thermal intermediate.

The thermal unfolding curves of the WT protein are shown in Figure 4A. In agreement with our hypothesis, the 11 individual single mutants of the wild type protein present (Table 1 and Figure 5A) increased *T_1_* values (from 2.6 to 8.8°C) relative to WT (average Δ*T_1_* = 6.4°C). Also in agreement with our hypothesis, their *T_2_* values were not generally increased relative to WT. Instead, one mutant displayed a lower *T_2_*, two mutants displayed essentially the same *T_2_* as WT, seven mutants showed modest increases and only one mutant showed a significant increase (average Δ*T_2_* = 2.2°C). The feasibility of specifically increasing *T_1_* in three-state proteins by introducing common types of stabilizing mutations in the less stable region of the protein or at its interface with the stable region is thus clearly demonstrated.

On the other hand, the fact that introducing stabilizing mutations in the stable region of the protein only serves to increase *T*_2_ had been amply demonstrated in previous work using charge reversal mutations^15^. Indeed, the four more successful such mutations, E20K, E40K, E72K and D75K, lead to average Δ*T_2_* = 5.2°C with a negligible effect in the relevant stability (average Δ*T_1_* = 0.1°C; Figure 5C). We have combined two of these mutations at the stable region (E20K and E72K, Table 1) with mutation D126K at the less stable one to build a second reference protein termed 3M apoflavodoxin. 3M apoflavodoxin displays a CD spectrum and an enthalpy change for the first unfolding equilibrium (Δ*H_1_*) similar to those of WT apoflavodoxin (Figure 3 and Table 2) while its Δ*H_2_* is larger, as expected from its higher *T_2_*. Therefore, the overall structures of the 3M and of the WT reference proteins are likely similar. Relative to WT, 3M display a moderately increased *T_1_* (by 7.2°C) and an even greater increase in *T_2_* (by 11.6°C). These increases in melting temperature values are close to accumulative of the individual effects exerted in WT by the three mutations in 3M. As a consequence, the temperature gap (*T_2_*–*T_1_*) is larger in 3M than in WT, which offers more room for increasing the relevant stability (Table 2). 3M apoflavodoxin thus allows to retest in the context of a more stable protein whether specific stabilization of the less stable region is the way to specifically increasing *T_1_*. Nine of the individual mutations tested in WT (see above) have also been introduced in 3M (Table 2) and their stabilizing effects have been determined (Table 2). Once again, in agreement with our hypothesis, all 3M-based single mutant proteins but one (3M/I92A) present significantly increased *T_1_* values (from 5.0 to 18.0°C) relative to 3M (average Δ*T_1_* = 9.0°C; Figure 5B) while their *T_2_* values are only slightly higher than that of 3M (average Δ*T_2_* = 1.3°C).

### Additivity of mutations

Rationally designed mutations, such as those introduced in this work, tend to yield moderate increases in *T_m_* values. The single mutations tested on the less stable region of either WT or 3M apoflavodoxin have each increased the *T_1_* of the corresponding reference protein by 6–8°C. If their effects could be accumulated, larger thermostabilizations would be feasible. We first tried accumulating increases in *T_1_* on the WT/92A mutant scaffold but only one out of three single point mutations added (mutant WT/92A/142V) significantly increased *T_1_* (Table 1). Thus, we tried increasing *T_1_* from WT/142V. Besides 92A, three other mutations: 59A, 87V or 100N were individually introduced on WT/142V (Table 1). In this case, the increases in *T_1_* brought about by those four mutations on the WT/142V protein were similar to those previously observed on WT (Δ*T_1_* = 7.7, 7.3, 8.3 or 8.8°C, respectively in WT versus 7.2, 6.9, 9.9, or 9.1°C on the already stabilized WT/142V). Therefore, each of those four variants accumulated the increase in *T_1_* caused by mutation 142V (4.1°C) with those of the particular additional mutation, elevating the WT *T_1_* of 42.8 to around 55°C. At the same time, the accumulation of stabilizing mutations in the less stable region begun to significantly increase the stability of the stable domain, so that the four variants exhibited *T_2_* values around 64°C, well above the *T_2_* of 55.1°C in WT.

To assess whether a much more stable apoflavodoxin variant could similarly benefit from accumulative stabilization of *T_1_*, we also combined *T_1_*-increasing mutations on 3M (table 2). As with WT, we first tried accumulating mutations on the 3M/92A variant. Four *T*_1_ stabilizing point mutations were tested (59A, 87V, 100N and 142V) and all of them further increased *T_1_* by an average of 10.7°C, quite similar to their average stabilizing effect of 12.6°C previously observed on 3M. We also tried accumulating mutations on a different 3M scaffold: 3M/142V. In addition to the 92A mutation already discussed, two other mutations (87V and 100N) were individually added to such a scaffold. In either case, the stabilization achieved was very large and a change from three-state to two-state unfolding behavior was observed (Table 2). On average, the six variants discussed, each accumulating two stabilizing mutations on 3M, raised *T_1_* from 50.0°C in 3M to around 65.0°C. In these six variants, the stabilization of the second transition was smaller, as expected, but significant: from a *T_2_* of 66.7°C in 3M to an average of 71.1°C. It seems clear that the relevant stability of both WT and of its more stable 3M version can be easily increased by accumulating the *T_1_*-increasing effects of individual mutations.

### Thermostabilization versus global stabilization determined by chemical denaturation

**Figure 6:**
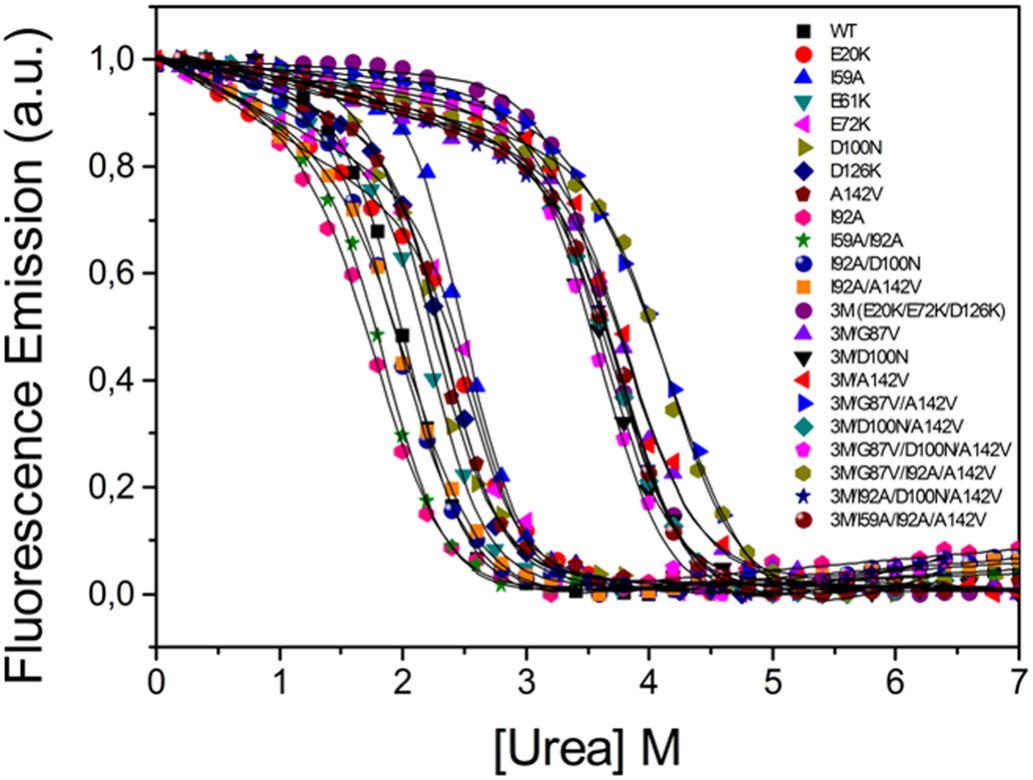
Chemical unfolding curves of apoflavodoxin and variants thereof, followed by fluorescence emission at 320 nm. The curves are roughly normalized to signal spans from 0 to 1 and do not represent fractions of folded protein. The solid lines are fits to a two-state equation.

Chemical denaturation plus LEM analysis^42^ is a common way to accurately determine folding free energy differences (i.e. protein thermodynamic stability) at a given temperature below that of thermal denaturation. Using chemical denaturation, the difference in stability of protein variants can be readily obtained. One question here is whether chemical denaturation constitutes a reliable guide for the rational thermostabilization of complex proteins, and there are at least two reasons why it is not. The first reason, already explained, is that some proteins exhibit two-state chemical denaturation but three-state thermal denaturation. In such proteins an increase in the overall stability detected in chemical denaturation can translate into just an increase in the residual stability of the intermediate (Δ*G_IU_*) and leave the relevant stability of the native state (Δ*G_NI_*) unchanged. The second reason, that protein stability is a complex function of temperature, applies even when the stabilizing mutation is specifically introduced in the less stable region of a three-state protein. To illustrate these facts, we have determined by urea denaturation the global stability at 25.0°C of some of the flavodoxin variants described above (Figure 6 and Tables 3 and 4). While many of the point mutants of the WT and of the 3M reference apoflavodoxins displaying either an increased *T_1_* or *T_2_* are indeed more stable towards the full two-state unfolding (N-to-U) at 25°C probed by chemical denaturation (Tables 3 and 4; Figure 6), some others are not more stable or are even less stable. The discrepancy is particularly noticeable for the more thermostable 3M-based mutants. These results illustrate that although thermostabilization often converts into a greater stability at lower temperatures, selecting mutants for their greater stability at a low temperature is not a reliable criterion to guide rational thermostabilization.

### From three-state to two-state unfolding as the relevant stability is increased

**Figure 7:**
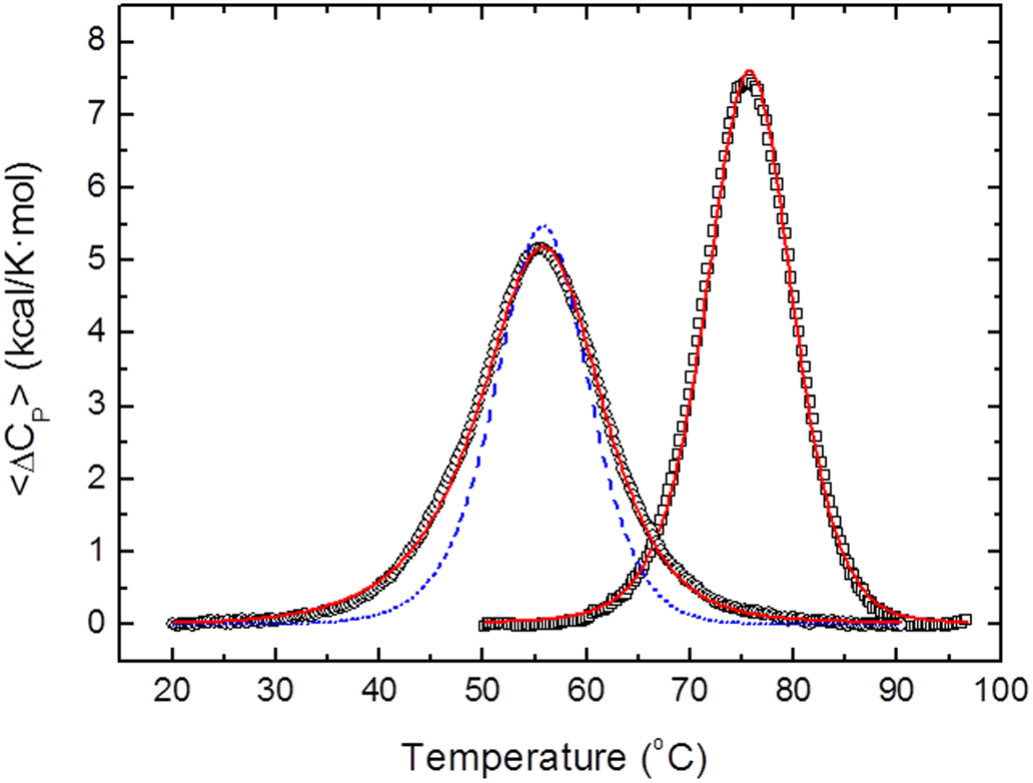
Differential scanning calorimetry (DSC) thermograms representing excess heat capacity *versus* temperature for wild type apoflavodoxin and the 3M/A142VG87VD100N mutant. The continuous red lines are the best fits of the wild type unfolding to a three-state model and that of the 3M/A142V/G87V/D100N unfolding to a two-state one. The discontinuous line in blue represents a two-state fit of the wild type protein, which is shown to illustrate that its thermal unfolding, unlike that of the 3M/A142V/G87V/D100N mutant, does not follow the simple two-state model.

The spectroscopic and thermodynamic properties of WT apoflavodoxin make the global fitting of several thermal unfolding curves monitored using different spectroscopic techniques the best way to accurately determine the two melting temperatures of its three-state equilibrium^15^. Due to the low enthalpy change of the first unfolding transition, accurate determination of *T_1_* by differential scanning calorimetry (DSC) is difficult. Still, DSC is the best way to unequivocally determine, from a comparison of the van't Hoff and calorimetric enthalpies^56^, whether the thermal unfolding of a specific flavodoxin variant retains the three-state character of the WT or has changed to two-state. For this reason the 36 flavodoxin variants described so far were routinely analyzed by DSC to confirm that they were indeed three-state mutants. Out of the 36 variants, 32 were clearly three-state. Two possible exceptions were 3M/D100N and 3M/A142V, for which reasonable two-state DSC fits could be obtained (Table 2). The apparent two-state behavior of these 3M-based mutants prompted us to analyze a second round of mutants introduced into 3M/A142V and we found two more mutants (3M/A142V/G87V and 3M/A142V/D100N) whose DSC profiles were best fitted to a simple 2-state unfolding (Table 2). The realization that, in those four mutants, *T_1_* had been raised to equal their corresponding *T_2_*, made us further combine stabilizing mutations. Thus, we introduced additional combinations of two stabilizing mutations (chosen between G87V, D100N, I59A and/or I92A) into 3M/A142V containing scaffolds and obtained four new mutants: 3M/A142V/G87V/I92A, 3M/IA142V/92A/D100N, 3M/A142V/I59A/I92A and 3M/A142V/G87V/D100N. For those four final mutants, the DSC thermograms (see Figure 7 for mutant 3M/A142V/G87V/D100N) clearly indicated that the thermal unfolding was two-state, with ratios of van't Hoff and calorimetric enthalpies between 0.96 and 1.1. Similarly, their spectroscopic curves could be superimposed and globally fitted to a two-state equation (see Figure 4B for mutant 3M/A142V/G87V/D100N).

Therefore sufficient accumulations of stabilizing mutations in the less stable region and/or in its interface with the stable one can in the end strengthen protein folding cooperativity to the point of transforming a three-state protein into a two state one. The prize is of course that, in the process, *T_1_* is so much raised that the increase in the relevant stability is very large. The average single *T_m_* of the last four two-state mutants discussed is of 72.6°C which, compared to the WT *T_1_* of 42.8°C, means the mutants have increased their relevant thermostability by 29.9°C. The most stable mutants, 3M/A142V/G87V/D100N and 3M/I92A/A142V/G87V, with *T_m_* of 74.9°C have been stabilized by 32.1°C, relative to WT. In terms of free energies, the increase in the relevant stability of the two more stable mutants from that of WT can be calculated using eq. 3 to be of around 8 kcal/mol.

### Divide and combine: a general strategy for the thermal stabilization of complex proteins

Based on the above results, we propose that efficient, relevant thermostabilization of three-state proteins can be achieved applying a “divide and combine” approach consisting of the following steps:

- 1-Structural delineation of the more stable and less stable regions (by either experiment^33^,^34^,^35^ or computation^36^,^55^).
- 2-Design, testing and selection of stabilizing mutations involving substitution of residues at the less stable region or at the interface between regions: type 1 mutations.
- 3-Design, testing and selection of stabilizing mutations involving substitution of residues at the stable region: type 2 mutations.
- 4-Combination of type 2 mutations to obtain a scaffold with higher *T_2_.*
- 5-Sequential combination of type 1 mutations on the previous scaffold.

A simpler method is possible by omitting steps 3 and 4 and directly combining type 1 mutations on the wild type protein, but a lower thermostabilization is to be expected in that case. It should be mentioned that, although stabilization of the less stable region may provide by itself the interface stabilization required to eventually obtain two-state full cooperative unfolding, a direct intervention including the introduction of a few stabilizing mutations at the interface seems advisable. Importantly, when a two-state variant is finally obtained, the target for thermostabilization is no longer confined to the small, less stable structural region, and the much larger mutational space of the entire protein becomes usable to proceed with further stabilization though rational design.

We would like to recall that proteins can also be stabilized through ligand binding, an important emerging field in protein formulation^57^,^58^ and in the development of a family of drugs known as pharmacological chaperones^59^,^60^. The underlying ideas of the divide and combine approach illustrated here with thermostabilization by site-directed mutagenesis might also be useful for increasing protein stability through ligand binding in complex proteins. The goal in that case would be the identification of ligands that specifically bind to the less stable region of the protein or at its interface with a more stable one.

In summary, the strategy proposed here is general and it only requires two things: the protein structure and information about which region is unfolding first.

## Author Contributions

E.L. prepared most protein variants and performed the corresponding experiments and analysis. I.C. prepared some protein variants and performed the corresponding experiments and analysis. M.B. designed some protein variants and performed experiments in an early stage of the work. A.V.-C. analyzed results and contributed to writing the manuscript. J.S. conceived the work, analyzed the results and wrote the manuscript. All authors reviewed the manuscript.

## Figures and Tables

**Table 1:** Thermal unfolding of apoflavodoxin variants: wild type (WT) and mutants thereof

Protein variant	Δ*H*_NI_ (kcal/mol)	*T_m_*_NI_ (°C)	Δ*H*_IU_ (kcal/mol)	*T*_mIU_ (°C)
**WT**	34.7 ± 2.0	42.8 ± 0.3	55.6 ± 2.0	55.1 ± 0.4
**I59A**	47.2 ± 0.1	50.1 ± 0.1	50.2 ± 0.5	55.3 ± 0.1
**E61K**	41.0 ± 0.9	47.8 ± 0.1	38.0 ± 1.0	58.6 ± 0.2
**G87V**	38.8 ± 0.7	51.1 ± 0.7	52.8 ± 2.3	61.1 ± 0.3
**I92A**	29.7 ± 6.9	50.5 ± 1.5	38.9 ± 2.9	53.2 ± 0.8
**Q99A**	52.1 ± 0.6	49.4 ± 0.1	56.2 ± 1.2	58.3 ± 0.4
**D100N**	47.0 ± 1.8	51.6 ± 0.5	53.3 ± 6.9	58.6 ± 0.9
**S121P**	33.4 ± 0.4	45.4 ± 0.3	64.1 ± 1.7	56.6 ± 0.1
**D126K**	40.6 ± 0.2	48.8 ± 0.2	45.1 ± 1.3	57.7 ± 0.1
**S129A**	40.7 ± 1.3	49.2 ± 0.2	48.8 ± 2.2	58.6 ± 0.3
**A142V**	33.6 ± 3.3	46.9 ± 0.7	57.3 ± 5.3	57.5 ± 0.6
**R155L**	34.3 ± 2.3	50.3 ± 0.9	42.4 ± 3.7	55.2 ± 0.7
**E20K**^a^	30.2 ± 0.3	42.7 ± 0.4	66.1 ± 1.3	59.7 ± 0.1
**E72K**^a^	33.2 ± 0.2	45.4 ± 0.3	57.8 ± 1.0	63.1 ± 0.1
**I92A/I59A**	34.3 ± 3.1	49.4 ± 1.0	61.6 ± 3.8	56.5 ± 0.7
**I92A/D100N**	48.3 ± 4.3	50.1 ± 0.6	60.1 ± 3.8	58.0 ± 0.6
**I92A/A142V**	40.4 ± 2.8	54.1 ± 1.7	48.1 ± 3.2	63.2 ± 3.8
**A142V/I59A**	36.4 ± 2.9	53.8 ± 0.8	61.6 ± 6.3	62.0 ± 0.8
**A142V/G87V**	37.4 ± 2.0	56.8 ± 0.6	42.4 ± 3.3	67.9 ± 1.5
**A142V/D100N**	39.4 ± 2.0	56.0 ± 0.5	47.3 ± 3.4	62.9 ± 0.5

^a^Mutations at the stable structural region. From Campos et. al, 2004^15^.

**Table 2:** Thermal unfolding of apoflavodoxin variants: 3M (WT plus E20K/E72K/D126K) and mutants thereof

	Global spectroscopic three-state fit				DSC two-state fit			
Protein variant	Δ*H*_NI_ (kcal/mol)	*T_m_*_NI_ (°C)	Δ*H*_IU_ (kcal/mol)	*T*_mIU_ (°C)	Δ*H*_ND_ (kcal/mol)	*T_m_*_ND_ (°C)	Δ*H*_VH_^a^ (kcal/mol)	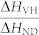
**3M**^b^	38.3 ± 2.8	50.0 ± 0.4	71.9 ± 2.4	66.7 ± 0.2	83.4 ± 0.2	67.7 ± 0.1	70.9 ± 0.3	0.85
**3M/I59A**	37.2 ± 1.1	57.6 ± 0.2	87.6 ± 2.4	68.0 ± 0.2	-	-		
**3M/E61K**	40.9 ± 0.2	55.0 ± 0.1	83.6 ± 2.3	67.3 ± 0.8	-	-		
**3M/G87V**	40.1 ± 1.2	58.8 ± 0.2	80.2 ± 0.4	68.8 ± 0.1	-	-		
**3M/I92A**	34.2 ± 2.0	50.6 ± 3.1	65.8 ± 0.7	65.1 ± 1.6	-	-		
**3M/Q99A**	47.9 ± 0.6	58.3 ± 0.1	81.9 ± 1.8	68.6 ± 0.1	-	-		
**3M/D100N**	49.1 ± 3.2	68.0 ± 0.1	95.9 ± 1.7	69.7 ± 1.1	71.4 ± 0.2	69.2 ± 0.1	67.0 ± 0.3	0.94
**3M/S121P**	40.8 ± 0.1	56.2 ± 0.1	75.3 ± 1.1	68.6 ± 0.1	-	-		
**3M/D129A**	55.1 ± 0.9	60.7 ± 0.3	83.4 ± 1.1	68.1 ± 0.1	-	-		
**3M/A142V**	19.7 ± 2.1	66.0 ± 4.7	83.7 ± 2.8	68.2 ± 0.4	72.1 ± 0.2	69.4 ± 0.1	75.4 ± 0.2	1.05
**3M/I92A/I59A**	46.5 ± 1.8	63.9 ± 0.3	73.7 ± 2.0	69.9 ± 0.8	-	-		
**3M/I92A/G87V**	52.4 ± 6.6	58.9 ± 1.8	37.1 ± 3.3	75.1 ± 9.5	-	-		
**3M/I92A/D100N**	46.5 ± 2.2	63.2 ± 0.6	96.9 ± 6.2	69.3 ± 0.5	-	-		
**3M/I92A/A142V**	43.6 ± 2.9	59.0 ± 0.8	56.5 ± 6.9	67.3 ± 1.9	-	-		
**3M/A142V/G87V**	-	-	-	-	76.7 ± 0.1	74.4 ± 0.1	79.8 ± 0.2	1.04
**3M/A142V/D100N**	-	-	-	-	82.0 ± 0.1	70.4 ± 0.1	83.6 ± 0.2	1.02
**3M/A142V/G87V/I92A**	-	-	-	-	79.1 ± 0.1	74.9 ± 0.1	75.9 ± 0.2	0.96
**3M/A142V/I92A/D100N**	-	-	-	-	82.3 ± 0.1	70.6 ± 0.1	85.6 ± 0.2	1.04
**3M/A142V/I92A/I59A**	-	-	-	-	87.6 ± 0.1	70.3 ± 0.1	92.9 ± 0.2	1.06
**3M/A142V/G87V/D100N**	-	-	-	-	84.6 ± 0.1	74.9 ± 0.1	84.5 ± 0.2	1.00
**WT**^b^					84.4 ± 0.2	55.8 ± 0.1	51.5 ± 0.2	0.61

^a^Van't Hoff enthalpies, Δ*H*_VH_, have been determined by DSC, assuming a two-state protein unfolding mechanism, using the known equation: 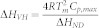, where *T*_m_ is the mid-transition temperature, *C*_p,max_ is the maximal value of the excess molar unfolding heat capacity, and Δ*H*_ND_ is the calorimetric enthalpy. If the assumed two-state unfolding model is correct the calorimetric enthalpy, Δ*H*_ND_, and the van't Hoff enthalpy, Δ*H*_VH_ are equal (i.e. Δ*H*_VH_/Δ*H*_ND_ = 1.0). Otherwise, both enthalpies are significantly different.

^b^The Δ*H*_VH_/Δ*H*_ND_ ratio of mutant 3M indicates its thermal unfolding is not two-state. The same applies to the WT protein included in this Table for comparison.

**Table 3:** Chemical unfolding of apoflavodoxin variants: wild type (WT) and mutants thereof^a^

Protein variant	*m* (kcal mol^−1^ M^−1^)	[urea]_m_ (M)	Δ*G* (kcal mol^−1^)	Δ[urea]_m_ (M)	ΔΔ*G*_ND_ (kcal mol^−1^)
**WT**	2.48 ± 0.05	2.07 ± 0.01	5.13 ± 0.02	-	-
**I59A**	2.50 ± 0.08	2.48 ± 0.01	6.19 ± 0.17	−0.40	−1.05 ± 0.17
**D100N**	2.26 ± 0.14	2.25 ± 0.02	5.08 ± 0.10	−0.18	0.06 ± 0.11
**A142V**	2.47 ± 0.14	2.31 ± 0.02	5.70 ± 0.24	−0.23	−0.56 ± 0.24
**I92A**	2.06 ± 0.10	1.82 ± 0.03	3.75 ± 0.04	0.25	1.39 ± 0.04
**E61K**^b^	2.66 ± 0.09	2.24 ± 0.01	5.96 ± 0.20	−0.17	−0.83 ± 0.20
**E20K**^b^	2.58 ± 0.07	2.56 ± 0.01	6.60 ± 0.18	−0.49	−1.47 ± 0.18
**E72K**^b^	2.40 ± 0.13	2.64 ± 0.02	6.34 ± 0.35	−0.57	−1.21 ± 0.35
**D126K**^b^	2.58 ± 0.10	2.35 ± 0.01	6.06 ± 0.24	−0.28	−0.93 ± 0.24
**I92A/I59A**	2.30 ± 0.09	1.90 ± 0.01	4.35 ± 0.03	0.18	0.79 ± 0.04
**I92A/D100N**	2.01 ± 0.08	1.98 ± 0.02	3.98 ± 0.01	0.09	1.16 ± 0.03
**I92A/A142V**	1.86 ± 0.07	2.06 ± 0.02	3.81 ± 0.11	0.02	1.32 ± 0.12

^a^The reported values of m, [urea]_m_ and Δ*G* are means of two determinations ± SE. Δ[urea]_m_ are differences between WT and mutant. Δ*G* are free energy differences of full unfolding (N-to-U) at 25.0°C. ΔΔ*G*_ND_ are differences between WT and mutant with propagated errors.

^b^From Campos et al, 2004^15^.

**Table 4:** Chemical unfolding of apoflavodoxin variants: 3M (WT plus E20K/E72K/D126K) and mutants thereof^a^

Protein variant	m (kcal mol^−1^ M^−1^)	[urea]_m_ (M)	Δ*G* (kcal mol^−1^)	Δ[urea]_m_ (M)	ΔΔ*G*_ND_ (kcal mol^−1^)
**3M**	2.30 ± 0.06	3.68 ± 0.01	8.46 ± 0.04	---	---
**3M/I92A**	1.93 ± 0.10	3.31 ± 0.02	6.40 ± 0.10	0.37	2.06 ± 0.11
**3M/G87V**	2.16 ± 0.07	3.86 ± 0.01	8.33 ± 0.16	−0.18	0.14 ± 0.16
**3M/A142V**	2.36 ± 0.09	3.84 ± 0.01	9.07 ± 0.57	−0.16	−0.60 ± 0.57
**3M/D100N**	2.16 ± 0.09	3.60 ± 0.02	7.78 ± 0.35	0.08	0.69 ± 0.35
**3M/I92A/G87V**	1.76 ± 0.07	3.65 ± 0.02	6.40 ± 0.24	0.04	2.07 ± 0.24
**3M/I92A/D100N**	1.90 ± 0.08	3.40 ± 0.01	6.46 ± 0.01	0.28	2.00 ± 0.03
**3M/I92A/A142V**	2.15 ± 0.10	3.68 ± 0.02	7.90 ± 0.13	0.01	0.56 ± 0.13
**3M/A142V/G87V**	1.68 ± 0.06	4.20 ± 0.02	7.05 ± 0.30	−0.52	1.42 ± 0.30
**3M/A142V/D100N**	2.00 ± 0.07	3.77 ± 0.01	7.53 ± 0.29	−0.09	0.93 ± 0.29
**3M/A142V/G87V/D100N**	1.88 ± 0.04	3.59 ± 0.02	6.72 ± 0.08	0.10	1.74 ± 0.09
**3M/I92A/A142V/G87V**	1.99 ± 0.07	4.16 ± 0.02	8.27 ± 0.17	−0.48	0.20 ± 0.17
**3M/I92A/A142V/D100N**	2.15 ± .070	3.83 ± 0.01	8.21 ± 0.17	−0.15	0.25 ± 0.17
**3M/I92A/A142V/I59A**	2.14 ± 0.08	3.78 ± 0.01	8.06 ± 0.01	−0.10	0.41 ± 0.02

^a^The reported values of m, [urea]_m_ and Δ*G* are means of two determinations ± SE. Δ[urea]_m_ are differences between WT and mutant. Δ*G* are free energy differences of full unfolding (N-to-U) at 25.0°C. ΔΔ*G*_ND_ are differences between WT and mutant with propagated errors.
